# Maximum independent sets of commuting and noninterfering inversions

**DOI:** 10.1186/1471-2105-10-S1-S6

**Published:** 2009-01-30

**Authors:** Krister M Swenson, Yokuki To, Jijun Tang, Bernard ME Moret

**Affiliations:** 1 Laboratory for Computational Biology and Bioinformatics, School of Computer and Communication Sciences, Ecole Polytechnique Fédérale de Lausanne, Switzerland; 2 Department of Computer Science and Engineering, University of South Carolina, Columbia, SC 29208, USA; 3 Swiss Institute of Bioinformatics, Lausanne, Switzerland

## Abstract

**Background:**

Given three signed permutations, an inversion median is a fourth permutation that minimizes the sum of the pairwise inversion distances between it and the three others. This problem is NP-hard as well as hard to approximate. Yet median-based approaches to phylogenetic reconstruction have been shown to be among the most accurate, especially in the presence of long branches. Most existing approaches have used heuristics that attempt to find a longest sequence of inversions from one of the three permutations that, at each step in the sequence, moves closer to the other two permutations; yet very little is known about the quality of solutions returned by such approaches.

**Results:**

Recently, Arndt and Tang took a step towards finding longer such sequences by using sets of commuting inversions. In this paper, we formalize the problem of finding such sequences of inversions with what we call signatures and provide algorithms to find maximum cardinality sets of commuting and noninterfering inversions.

**Conclusion:**

Our results offer a framework in which to study the inversion median problem, faster algorithms to obtain good medians, and an approach to study characteristic events along an evolutionary path.

## Background

The ordering and strandedness of genes on each chromosome of many organisms are now available, with many more to be added in the near future. Using this information, one can represent a genome as a collection of chromosomes, each of which is a linear or circular sequence of gene identifiers. Variations in the placement of the same genes, as well as variation in gene content and multiplicity, among organisms can then be analyzed. This data is of great interest to evolutionary biologists (and has been for quite some time: see [1]), but also to comparative genomicists (see, e.g., [2,3]) and to any researcher interested in understanding evolutionary changes in pathogens. Even when the data are restricted to singleton gene families (that is, when duplication and loss mechanisms are ignored), the resulting *gene-order *data have proved very useful in the analysis of small genomes (such as organelles) and in comparative genomics. In the past ten years, there has been a large increase in work done on analyzing such data, gene-order data in particular (see, e.g., [4]). Evolutionary biologists have sought to exploit the advantages of gene-order data (no need for reconciliation of gene trees, very little saturation, existence of rare events that uniquely characterize some very old divergences, etc.), but have had to contend with the high computational complexity of working with such data.

Of particular interest in a phylogenetic context is the problem of finding the median of three genomes, that is, finding a fourth genome that minimizes the sum of the pairwise distances between it and the three given genomes [5]. This problem, while being fairly easy for aligned sequence data, is NP-hard for gene-order data [6,7]. Since phylogenetic reconstruction based on reconstructing ancestral states may need to compute such medians repeatedly, fast approximations or heuristics are usually needed, although exact methods have done well for small genomes (from organelles, for instance) [8,9]. One such heuristic, implemented in the popular software MGR [10], attempts to find a longest sequence of inversions from one of the three given genomes that, at each step in the sequence, moves closer to the other two genomes. However, nothing is known about the theoretical behavior of this heuristic and no systematic experimental investigation of its usefulness has been conducted. Experimental evidence indicates that it leads to worse trees than an optimal median-solver [11], at least on small genomes, perhaps because the MGR search is limited to a small subset of possible paths. Recently, Arndt and Tang [12] provided significant improvement on this heuristic by considering sets of *commuting *inversions, that is, inversions that can be arbitrarily reordered among themselves without affecting the end result; using a somewhat different framework, Bernt *et al*. [13] proposed an approach that is also based on such inversions.

In this paper, we show that finding maximum cardinality sets of commuting inversions is equivalent to finding maximum independent sets on circle graphs and so can be done in low polynomial time–we give a simple algorithm for this purpose. We also shed light on the relationship between maximal sets of noninterfering inversions and independent sets on circle graphs. We further classify sets of commuting inversions into *interfering *and noninterfering inversions, where *noninterfering inversions *are commuting inversions that also make maximal progress (e.g., towards a median), and introduce the notion of an inversion *signature*, which captures the unique rearrangements common to all sorting paths. Finally, we characterize the relationship of sets of noninterfering inversions to signatures and that of signatures to inversion medians.

For most of the paper, we show how to analyze single permutations in terms of commuting and noninterfering inversions; in later sections, we show how to extend the analysis to multiple permutations.

### Commuting and noninterfering inversions

**Figure 1:**
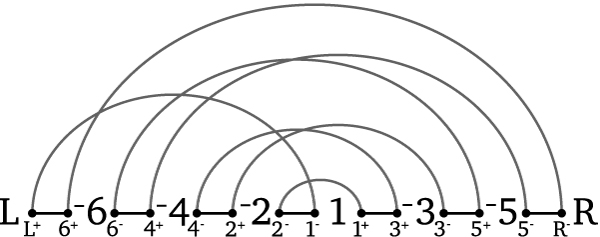
**An example of breakpoint graph**. *G*(*π *= -6 -4 -2 1 -3 -5). Black edges are reality edges and gray edges are desire edges.

An *inversion ρ*(*i*, *j*) transforms permutation *π *= *π*_1 _⋯ *π*_*i*-1_*π*_*i*_⋯*π*_*j*-1_*π*_*j*_*π*_*j*+1_⋯*π*_*n*_ into permutation *π*' = *π*_1_⋯*π*_*i*-1_*π*_*j*_*π*_*j*-1_⋯*π*_*n*_. Thus, the *inversion distance problem *between *π *and *τ *refers to finding a minimum series of inversions *ρ*_1_, *ρ*_2_,⋯, *ρ*_*t *_so that *π*·*ρ*_1_·*ρ*_2_⋯*ρ*_*t *_= *τ*. Because any series of inversions that sorts permutation *τ*_1 _to some permutation *τ*_2 _will also sort $\tau_{1}\tau_{2}^{-1}$ to the *identity *1 2 3 4 ⋯ *n*, we often only consider one permutation *π *= $\tau_{1}\tau_{2}^{-1}$ and call *d*(*π*) = *t *the *inversion distance*. Hannenhalli and Pevzner [14] showed how to use a graph representation of the two permutations, henceforth referred to as the *HP-graph*. An element *π*_*i *_is represented by vertices $\pi_{i}^{-}$ and $\pi_{i}^{+}$, where $\pi_{i}^{-}$ is to the left of $\pi_{i}^{+}$ if and only if *π*_*i *_is positive, and the permutation is bracketed by *L*^+ ^on the left and *R*^- ^on the right. *Reality *edges represent current adjacencies and so connect vertices from adjacent elements, while *desire *edges represent adjacencies for the sorted permutation (the identity) and so connect $\pi_{i}^{+}$ to $\pi_{i+1}^{-}$. Every vertex has degree two so that every vertex is part of a cycle; cycles that overlap when embedding on a line, with all desire edges on the same side of the line, are part of a *component*. Each reality edge on a cycle has a relative *direction *imposed by a tour of the cycle, carried out by noting in which direction the edges are traversed relative to the embedding. In Figure 1, edges (L^+^, 6^+^) and (2^-^, 1^-^) share a direction while all the others are of the opposite direction.

We say that inversion *ρ*(*i*, *j*) *acts upon *a reality edge e if e is either the *i*th or (*j *+ 1)st reality edge from the left; similarly, we say that inversion *ρ*(*i*, *j*) *acts upon *a desire edge *e *if *e *is incident on the rightmost vertex of the *i*th reality edge or on the leftmost vertex of the (*j *+ 1)st reality edge. In our example, the inversion over substring "-6 -4 -2 1 -3" (also known as *ρ*(1, 5)) acts upon reality edges (L^+^, 6^+^) and (3^-^, 5^+^). It acts upon desire edges (6^+^, R^-^) and (3^-^, 2^+^) and so affects vertices 6^+ ^and 3^-^. An *oriented *inversion acts upon reality edges from the same cycle of opposite direction. A component is oriented iff it has an oriented inversion that acts upon it, otherwise it is unoriented. A permutation that has at least one unoriented cycle has at least one *hurdle*, indicating that at least one additional inversion will be needed. See [14,15] for a more complete description.

Oriented inversions are of particular interest because, in the absence of hurdles, they are the only inversions that move *π *one inversion closer to the identity. A set of oriented inversions on a permutation *π commutes *iff the application of its inversions, in any order, yields the same final permutation.

**Definition 1**. *A set of m inversions on π *(*with respect to τ*) *is *noninterfering *if and only if*

1. *the set is commuting; and*

2. *applying these inversions in any order moves π closer to τ by m inversions*.

**Example 1**. *For π *= *-6 -4 -2 1 -3 -5 a maximum cardinality set of commuting inversions is *{*ρ*(1, 1), *ρ*(1, 4), *ρ*(1, 5), *ρ*(1, 6), *ρ*(2, 3), *ρ*(3, 3), *ρ*(4, 4)} *while a maximum cardinality set of noninterfering inversions is *{*ρ*(1, 1), *ρ*(1, 2), *ρ*(1, 4), *ρ*(4, 4)}.

### Inversion graphs and inversion signatures

*A sorting path *is a shortest sequence of oriented inversions on *π *with respect to some *τ*. The *inversion graph *is the graph of all sorting paths between *π *and *τ*; the permutations are vertices and edges link permutations that are one inversion away from each other.

**Definition 2**. *The intersection of all inversion graphs from a set of permutations P to permutation τ is the *inversion signature subgraph *and any vertex *(*permutation*) *in this subgraph is an *inversion signature.

A signature is *maximal *if there exists no neighbor to it in the signature subgraph that is farther from *τ*; a maximal signature that is as far from *τ *as any other is called a *maximum *signature.

**Figure 2:**
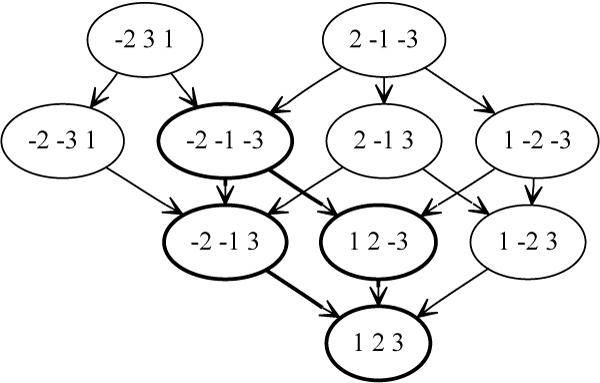
**The union of the inversion graphs for *P *= {-2 3 1, 2 -1 -3} and *τ *= 1 2 3**. The inversion signature subgraph for *P *is highlighted in bold.

**Example 2**. *In Figure *2* we have P *= {*2 -1 -3*, *-2 3 1 *} *and τ *= *1 2 3 *(*the *identity *permutation of length 3*). *The inversion signature subgraph is outlined in bold. The signatures in this case are -2 -1 -3*, *-2 -1 3*, *1 2 -3*, *and the trivial signature τ *= *1 2 3*. *The only maximum signature is also the only maximal signature -2 -1 -3*.

A set of noninterfering inversions of size *m *constitutes a subgraph of the signature subgraph of size $\sum_{i=0}^{m}\left(\begin{array}{c} m\\ i \end{array}\right)=2^{m}$. Siepel et al. [9] showed that a median for any signature between *τ *and *P *is also a median for *τ *and *P*.

### Circle graphs and permutation graphs

Consider drawing a set of chords with each endpoint of the chord on the same circle. The *circle graph *represents the intersection of these chords where each vertex corresponds to a chord and each edge corresponds to intersecting chords [16]. For a permutation we can define a *permutation graph *as follows. Each vertex is an element of the permutation and an edge (*u*, *v*) exists iff *v *> *u *and *v *appears to the left of *u *in the permutation [17]. Clearly, any permutation graph is a circle graph.

## Methods

### Maximum sets of commuting inversions

We now show how to find a maximum cardinality set of commuting inversions efficiently–omitting most proofs due to space limitations. We can interpret the indices of an inversion to be indices of an interval on a line. Two intervals are said to *overlap *if they share more than one point and neither is contained within the other.

**Lemma 1**. *A set C of inversions commutes if and only if no two inversions from C overlap*.

*Proof*. Assume the pair *a, b *∈ *C *overlaps. Fix an ordering of all inversions from *C *so that *b *is the last inversion and it immediately follows *a*. For this ordering the rightmost element of *a *will end up to the left of the leftmost element of *a*. Now take an ordering identical to the previous one but with *a *following *b*; this yields a contradiction because the rightmost element of *a *is right of the leftmost element of *a*. The other direction is trivial.   □

**Figure 3:**
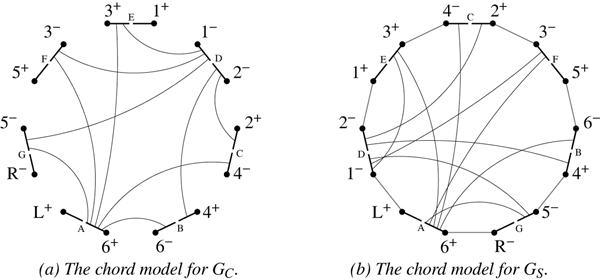
The chord models for circle graphs representing the constraints on G(*π *= -6 -4 -2 1 -3 -5).

We have a set of intervals that, when projected onto a circle, yields a chord model of a circle graph [18];

Figure 3(a) illustrates the concept. Call this circle graph *G*_*C*_; a maximum independent set of *G*_*C *_corresponds to a maximum independent set of commuting inversions. The HP-graph can be built in linear time [19] and a maximum independent set of *G*_*C *_can be computed in *O*(*n*^2^) time with the algorithm of Valiente [20]; we thus have the following theorem.

**Theorem 1**. *A maximum cardinality set of commuting inversions can be found in O*(*n*^2^) *steps*.

### Maximum sets of noninterfering inversions

Finding a set of noninterfering inversions is more demanding than finding a set of commuting inversions, but we can also use maximum independent sets in the circle graph–except that now we need to use the union of two circle graphs.

A set of noninterfering inversions is also a set of commuting inversions; but additional constraints must be introduced to ensure that the selected set of commuting inversions also sort the permutation. We now proceed to develop the theoretical background to represent these additional constraints by another circle graph, beginning with single-cycle components of the HP-graph and then extending the characterization to general components.

#### Single cycle components

One important property of commuting inversions is that the application of one inversion cannot alter the orientation of an inversion with which it commutes.

**Lemma 2**. *Given commuting oriented inversions ρ*(*i*, *j*) *and σ *(*k*, *l*), *the application of ρ will either make σ span two different cycles or leave σ oriented*.

*Proof*. Call *r *and *s *the reality edges acted upon by *σ*. At least one of *r *or *s *remains intact after the application of *ρ*, say *r*. At least one of the vertices incident to *s *must remain intact, say *v*. There is a path *P *from *v *to some *u *incident to *r *that does not include *r*. The adjacencies of *v *and *u *are not affected by *ρ*; moreover, because *σ *is oriented, if *v *is on some side of *s *then *u *is on the same side of *r*. But *ρ *can only remove a subpath of the cycle when creating another cycle. Because *ρ* and *σ *commute, *u *and *v *will remain on the same sides of their respective reality edges, thus leaving the inversion *σ *oriented.   □

Each oriented inversion will split the cycle by swapping the affected vertices of the desire edges acted upon. Thus, when we embed the cycle on a circle, we can represent the action of an inversion as a chord with its endpoints on those desire edges. For two inversions that intersect and act upon a disjoint set of desire edges, we know that applying one of them will put the reality edges acted upon by the other on different cycles; so in this case intersecting chords represent inversions that interfere.

Finding the interactions between inversions that share a reality edge is harder. Consider the set of inversions that share a reality edge as an endpoint and share the same desire edge; for example, the set of inversions that share reality edge (2^-^, 1^-^) is {*ρ*(2, 3), *ρ*(3, 3), *ρ*(4, 4), *ρ*(4, 5), *ρ*(4, 6)}, which can be partitioned into inversions that share (2^-^, 1^+^) {*ρ*(2, 3), *ρ*(3, 3)} and those that share (1^-^, L^+^) {*ρ*(4, 4), *ρ*(4, 5), *ρ*(4, 6)}. Let us order such a set *I *in two ways. The ordering *α*: *I *↦ ℕ numbers inversions from shortest to longest. There exists a vertex *v *that is affected by every inversion in the set (because of the sharing of edges); our second ordering *β*: *I *↦ ℕ numbers inversions by the order in which we visit the *other *("non-*v*") endpoint, starting at the common reality edge and proceeding through *v*.

**Lemma 3**. *Given inversions i*, *j *∈ *I*, *i interferes with j iff we have α*(*i*) > *α *(*j*) *and β *(*i*) <*β *(*j*).

In other words, an inversion interferes with all shorter inversions that appear after it on the cycle.

*Proof*. *v *is the shared vertex that is affected by all inversions in *I*. For an inversion *i *∈ *I *and any *j *∈ {*k*||*k *∈ *I*\{*i *} and *α *(*i*) > *α *(*k*)} with endpoints *v *and *u *respectively, *i *interferes with *j *iff *u *ends up on a different cycle than *v *after applying *i*. If we follow the cycle in the same order used to build *β*, the reality edges we visit before encountering *u *are those that remain on the cycle with *v *when it is attached by the new reality edge. So those inversions that act upon such reality edges remain oriented and they are exactly those *j *that have *β *(*j*) <*β *(*i*). The others will respect *β *(*i*) <*β *(*j*).   □

**Figure 4:**
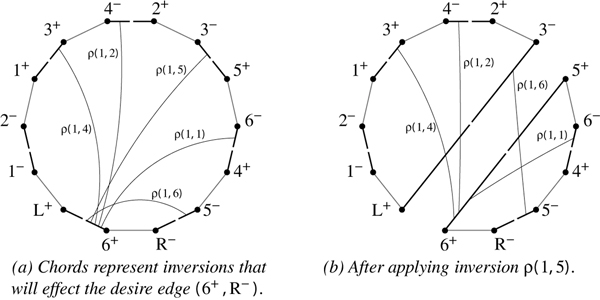
***G*(*π *= -6 -4 -2 1 -3 -5) embedded on a circle**. We see the affect that inversion *ρ*(1, 5) has on those inversions acting upon the same desire edge; *ρ*(1, 5) interferes with *ρ*(1, 2), *ρ*(1, 4), and *ρ*(1, 6), but not *ρ*(1, 1).

**Example 3**. *Figure *4(a)* shows the graph from Figure *1* embedded on a circle. α imposes the ordering on all inversions that share desire edge *(6^+^, R^-^) *so that α *(*ρ*(1, 1)) <*α *(*ρ*(1, 2)) <*α *(*ρ*(1, 4)) <*α *(*ρ*(1, 5)) <*α *(*ρ*(1, 6)). *We also have β *(*ρ*(1, 6)) <*β *(*ρ*(1, 1)) <*β *(*ρ*(1, 5)) <*β *(*ρ*(1, 2)) <*β *(*ρ*(1, 4)). *So for *(1, 5) *we have α *(*ρ*(1, 5)) > *α *(*ρ*(1, 4)) > *α *(*ρ*(1, 2)), *as well as β *(*ρ*(1, 5)) <*β *(*ρ*(1, 2)) <*β *(*ρ*(1, 4)), *which tells us that ρ*(1, 5) *interferes with ρ*(1, 2) *and ρ*(1, 4). *Further*, *α *(*ρ*(1, 5)) <*α *(*ρ*(1, 6)) *and β *(*ρ*(1, 5)) > *β *(*ρ*(1, 6)) *shows that ρ*(1, 5) *interferes with ρ*(1, 6). *Figure *4(b)* shows the result of applying inversion ρ*(1, 5) *on the graph*.

**Corollary 1**. *The interference relationship between all inversions that act on the same desire edge can be represented by a permutation graph*.

**Theorem 2**. *G_S _can be represented by a circle graph*.

*Proof*. If two inversions both act on a reality edge, then apply Corollary 1. Otherwise, embed the cycle on a circle and notice that the effect of an inversion is to split the circle (see Figure 4). A chord model representing the interference between two inversions that do not share a reality edge is obtained by drawing a chord for each inversion between the reality edges it acts upon.   □

Figure 3 shows the two circle graphs that represent the constraints of the HP-graph from Figure 1. In this case, *G*_*C *_is a subgraph of *G*_*S *_so *G*_*C *_∪ *G*_*S *_is a circle graph. A maximum cardinality set of noninterfering inversions would be represented by the set of chords {*AB*, *AC*, *AE*, *DE*} (matching that from Example 1).

The union of two circle graphs, however, need not yield a circle graph. To handle this issue, we decompose the instance into computationally easy and hard subinstances by using the first of two phases from the polynomial-time circle graph recognition algorithm of Bouchet [21,22]. This first phase repeatedly decomposes the graph by *join decomposition*; it finds a complete bipartite decomposition, call it *V*_1*c *_⊆ *V*_1 _and *V*_2*c *_⊆ *V*_2_, then replaces it by the two graphs induced by taking only vertices in *V*_1 _and *V*_2_, and adding a marker vertex to each graph connected to only *V*_1*c *_and *V*_2*c *_respectively. If such a decomposition does not exist, the subgraph at hand is said to be *prime*. In the second phase, a chord model is found for each prime subgraph. If every prime subgraph yields a chord model, then we can apply the quadratic-time algorithm of Valiente [20] to find the maximum independent set of the circle graph and we are done. If only some subgraphs yield a chord model, we can handle those independently with the same algorithm. Thus the computationally hard subgraphs are those prime subgraphs that do not yield a chord model; it is on these subgraphs that we are forced to run a general algorithm for maximum independent set.

**Figure 5:**
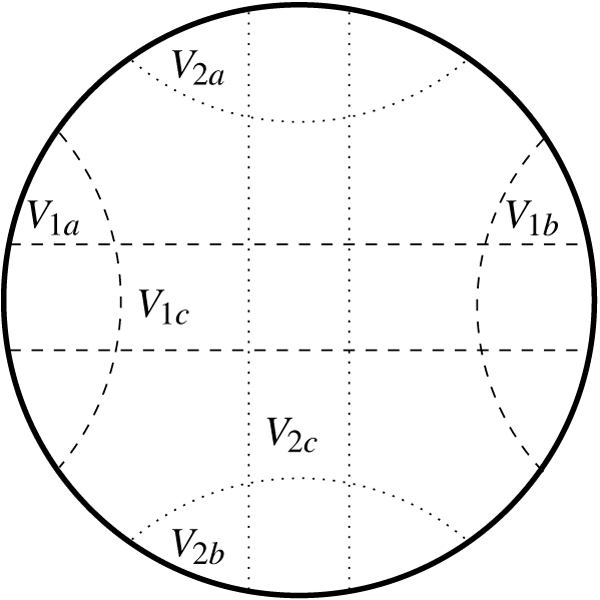
What the chord model of a join decomposition looks like if such a chord model exists.

Figure 5 shows how a set of vertices is partitioned into connected components *V*_1 _= *V*_1*a *_∪ *V*_1*b *_∪ *V*_1*c *_and *V*_2 _= *V*_2*a *_∪ *V*_2*b *_∪ *V*_2*c, *_where *V*_1*a*_, *V*_2*a*_, *V*_1*b*_, and *V*_2*b *_are possibly empty sets.

In our setting, the sets *V*_1*a*_, *V*_1*b*_, and *V*_1*c *_(resp. *V*_2*a*_, *V*_2*b*_, and *V*_2*c*_) may not actually yield chord models, but the representation of Figure 5 shows how the independent sets of such a decomposition interact with each other.

When composing solutions of independent sets on hard subgraphs, solutions we denote by *MIS*(·), we must consider two possibilities: (i) vertices from *V*_1*c *_and *V*_2*c *_are used for *MIS*(*V*_1_) and *MIS*(*V*_2_) respectively; or (ii) vertices from neither or only one of the two are used. In the later case vertices from both independent sets will be in the independent set for *G*_*S *_∩ *G*_*C*_. In the former case we can use the vertices from *V*_1*c *_or from *V*_2*c *_but not both, so we recursively test *MIS*(*V*_1*a *_∪ *V*_1*b*_) + *MIS*(*V*_2_) and *MIS*(*V*_2*a *_∪ *V*_2*b*_) + *MIS*(*V*_1_) and use the larger of the two as the score for the subproblem.

#### Multiple cycle instances

We now show how to transform a multiple cycle component into a single cycle while appropriately ignoring inversions that are created by the process.

Hannenhalli and Pevzner [14] introduced the notion of a (*g*, *b*)-*split *where a cycle of length six or larger is split by adding two vertices so as to preserve at least one minimum sorting path. Such a change in the graph can be represented in the corresponding permutation by a remapping of some vertex labels, a process called a (*g*, *b*)-*padding*. Here we introduce the inverse operation to the split, the (*d*, *r*)-*join*, which takes two cycles and joins them so as to preserve all sorting paths, along with an inverse analog to the padding, the (*d*, *r*)-*shrink*. A (*d*, *r*)-join removes the vertices *x*^- ^and *x*^+ ^(from two different cycles) for some permutation element *x *along with reality edges (*x*^-^, *r*_1_) and (*x*^+^, *r*_2_) and desire edges (*x*^-^, *d*_1_) and (*x*^+^, *d*_2_). The edges *r *= (*r*_1_, *r*_2_) and *d *= (*d*_1_, *d*_2_) are then added to form a valid HP-graph *Ǧ*(*π*). It is easy to verify that a (*d*, *r*)-join operation is equivalent to a (*d*, *r*)-shrink which acts by removing the element *x *and renaming all other elements with magnitude *i *> *x *to have magnitude *i *- 1 with the same sign. Hence we have *G*(πˇ) = *Ǧ*(*π*).

**Lemma 4**. *Apply to permutation π a *(*d*, *r*)-*shrink by removing an element x *(*corresponding to vertices x*^-^*and x*^+ ^*from two different cycles) to obtain *πˇ. *The inversion graph for π is a subgraph of the inversion graph for *πˇ*and d *(*π*) *equals d*(πˇ).

*Proof*. The length of the permutation decreases by one but so does the number of cycles, therefore we have *d*(*π*) = *d*(πˇ). We now show that the (*d*, *r*)-join of cycles *C*_1 _and *C*_2 _turning *G*(*π*) to *Ǧ*(*π*) will preserve the relative direction between edges. Fix a direction on the cycle with reality edge (*x*^-^, *r*_1_) by visiting *r*_1 _before *x*^- ^followed by *d*_1_. Similarly, fix a direction on the cycle with edge (*x*^+^, *r*_2_) by visiting *d*_2 _before *x*^+ ^followed by *r*_2_. Thus, after the application of the (*d*, *r*)-join the remaining reality edge *r *can be visited from *r*_1 _to *r*_2 _in a tour continuing to *d*_2 _and *d*_1 _from desire edge *d*. Since the direction for the new edges is consistent with the direction of the removed edges, the direction of *r *to reality edges in *C*_1 _and *C*_2 _is also consistent. So any inversion that acts on edges (*x*^-^, *r*_1_) or (*x*^+^, *r*_2_) for a sorting path on *π *will now act on *r *for a sorting path on πˇ. Since (*x*^-^, *r*_1_) and (*x*^+^, *r*_2_) are on different cycles of *G*(*π*), there can be no oriented inversions done that act on both at the same time.   □

An important corollary is that all oriented inversions on *π *will be preserved. Thus, we can shrink a multiple cycle component to an "equivalent" cycle and then run the algorithm while ignoring oriented inversions introduced by the shrinking process.

### Handling multiple permutations

When improving the MGR heuristic for medians or implementing a greedy heuristic for maximum signature computation, one needs to consider sets of inversions that occur in multiple permutations. This is done by simply ignoring intervals that do not occur as oriented inversions in all permutations, while merging the constraints on the remainder of the permutations. That is, to find the maximum independent set of commuting or noninterfering inversions on many permutations, take the intersection of the sets of oriented inversions over all permutations and run our algorithm on the union of the remaining constraints.

### Two notes on hurdles

Hurdles complicate our analysis in two places. First, while inversions that are unsafe on their own are easily identified and thus removed from consideration before running our algorithm, it is possible that a set of noninterfering inversions, each of which is safe by itself, can collude to create a hurdle. We can check for this problem, but the time requirements immediately increase as a result.

Second, a permutation that already contains many hurdles automatically yields a large (exponential in the number of hurdles) number of sorting paths, since hurdles can be merged two by two in almost every possible way (it suffices that the merged hurdles be nonadjacent, for instance). Each combination of hurdle merges yields a new set of oriented inversions, but it is not clear whether an exponential search of these combinations is necessary. Fortunately, hurdles are very rare in practice (for genomes subjected to rearrangements through inversions, at least) [23,24].

### A new median solver

We improved the MGR heuristic using maximum independent sets of noninterfering inversions. Given three genomes *G*_1_, *G*_2 _and *G*_3_, we define the median score of a genome *G *to be *d*(*G*, *G*_1_) + *d*(*G*, *G*_2_) + *d*(*G*, *G*_3_), where *d*(*G*, *G*_*i*_) is the distance between genome *G *and *G*_*i*_. To find the genome that minimizes the median score, the new median solver chooses the maximum independent set of inversions which brings *G*_1 _closer to both *G*_2 _and *G*_3_. The algorithm will then iteratively compute maximum independent sets of inversions in the three genomes until the maximum sets are empty. At the end of this procedure, the three given genomes are transformed to three (potentially) new genomes and we report the one with the lowest median score as the resulting median.

## Results and discussion

To assess the speed and accuracy of our new solver, we tested it using the same datasets used by Arndt and Tang [12]. These datasets were generated by assigning the identity permutation to the internal node and three leaves were created by applying rearrangement events along each edge. The number of events on each edge is a function of the total number of evolutionary events and of the tree shape. The total number of events used was in the range of 80 to 140 and three tree shapes were used: trees with edges of almost equal length; trees with one edge about twice longer than the other two; and trees with one edge about three times longer than the other two. We compared the new method to Caprara's median solver (exact but slow), to MGR, and to the solver of Arndt and Tang. For each combination of parameters, ten trees were generated and the average results are reported.

Table 1 and Table 2 show the median scores found by each method, and Table 3 and Table 4 show the time used by each method. Our new method not only runs significantly faster than MGR–when the datasets have many inversions, our new method is about 20 ~30 times faster than MGR–but it also returns more accurate medians. Our new method also improves on that of Arndt and Tang: it is from 3 to 100 times faster while never losing more than 1 ~2% in accuracy. The search strategies of these two solvers are different: our solver only searches maximum independent sets and will halt when the set is empty, while Arndt's solver uses a heuristic to decide the independent sets and will keep searching even when there is no independent set. Thus Arndt's solver is much slower but a bit more accurate than our new median solver. The accuracy of our new solver can be further improved with some additional computation. The three new genomes obtained when the search stops actually form a new instance of the median problem. We applied Caprara's solver to these new, smaller, median problems and found that the scores were improved for small to medium numbers of inversions–often to the point of matching the optimal solution. (For large numbers of inversions, however, the new median instances remained very difficult to solve exactly.)

**Table 1:** Comparison of median scores for *r *≤ 100

	(1:1:1)		(2:1:1)		(3:1:1)	
	
	r = 80	r = 100	r = 80	r = 100	r = 80	r = 100
Score lower bound	86.2	104.2	89.4	105.8	85.7	101.3

Caprara's median score	87.9	107.6	91.4	109.8	88.0	105.2

Arndt's median score	88.2	109.5	91.8	111.4	89.1	106.7

MGR median score	90.3	113.7	94.3	116.8	89.8	110.0

New method's median score	89.1	111.8	92.6	114.1	90.0	108.1

**Table 2:** Comparison of median scores for *r *≥ 120. N/A indicates a method cannot finish

	(1:1:1)		(2:1:1)		(3:1:1)	
	
	r = 120	r = 140	r = 120	r = 140	r = 120	r = 140
Score lower bound	116.1	123.5	116.1	122.7	110.3	117.6

Caprara's median score	N/A	N/A	N/A	N/A	N/A	N/A

Arndt's median score	125.8	135.3	124.5	134.7	117.9	127.0

MGR median score	132.9	143.6	131.4	142.8	123.6	135.1

New method's median score	127.9	139.5	126.9	138.5	120.6	130.1

**Table 3:** Comparison of running time for *r *≤ 100 (in seconds)

	(1:1:1)		(2:1:1)		(3:1:1)	
	
	r = 80	r = 100	r = 80	r = 100	r = 80	r = 100
Caprara's time	3.6	12876	57.2	31387	4.3	6908

Arndt's time	324	551	123	409	1.6	9.3

MGR time	11.2	51.9	11.6	78.2	10.3	35

New method's time	3.3	5.3	4.1	8.4	4.6	9.1

**Table 4:** Comparison of running time for *r *≥ 120 (in seconds)

	(1:1:1)		(2:1:1)		(3:1:1)	
	
	r = 120	r = 140	r = 120	r = 140	r = 120	r = 140
Caprara's time	> 172880	> 172880	> 172880	> 172880	> 172880	> 172880

Arndt's time	1485	1187	673	453	30	226

MGR time	271.6	560.1	237.8	626.9	135.3	385.4

New method's time	13.8	19.7	11.1	21.3	9.2	12.4

## Conclusion

We presented two new algorithms: a quadratic-time algorithm to compute a maximum set of commuting inversions and a more complex algorithm to compute a maximum set of noninterfering inversions. The latter algorithm can also run in quadratic time by using the circle graph recognition of Spinrad [25]–and the conditions under which this algorithm can be used are detectable in low polynomial time. When these conditions are not met, our algorithm decomposes the instance so that only certain subinstances require exponential work. It is worth noting that, due to the intersection step in our algorithm, the more genomes that are compared, the sparser the intersection will be and the faster the algorithm will run.

Arndt and Tang [12] showed that an MGR-style search for medians can be improved through a better choice of inversions; our new median solver, using the algorithm for computing a maximum set of noninterfering inversions, further improves on these results, both in terms of accuracy and in terms of speed. We expect further research into the relationship between inversion medians, signatures, and noninterfering inversions will uncover much more structure that can be used to design yet faster algorithms, thereby providing a practical tool for the reconstruction of ancestral genomes.

## Competing interests

The authors declare that they have no competing interests.

## Authors' contributions

KMS and BMEM contributed to the development and implementation of the algorithms, and YT and JT were in charge of developing the new median solver and conducting simulations.
